# Exploring the feasibility of using mobile phones to improve the management of clients with cervical cancer precursor lesions

**DOI:** 10.1186/s12905-018-0702-1

**Published:** 2019-01-07

**Authors:** Jennifer Moodley, Deborah Constant, Matthys H. Botha, Frederick H. van der Merwe, Amanda Edwards, Mariette Momberg

**Affiliations:** 1 Cancer Research Initiative, Faculty of Health Sciences, University of Cape Town, Anzio Road, Observatory, Cape Town, 7925 South Africa; 2 Women’s Health Research Unit, Faculty of Health Sciences, School of Public Health and Family Medicine, University of Cape Town, Anzio Road. Observatory, Cape Town, 7925 South Africa; 3 SAMRC Gynaecology Cancer Research Centre, Faculty of Health Sciences, University of Cape Town, Anzio Road. Observatory, Cape Town, 7925 South Africa; 4 Unit for Gynaecological Oncology, Tygerberg Academic Hospital and Stellenbosch University, Stellenbosch, South Africa

**Keywords:** mHealth, Cervical cancer, Screening, Pap smear, Colposcopy, Loss to follow-up, Prevention, eHealth

## Abstract

**Background:**

Cancer screening programs hold much potential for reducing the cervical cancer disease burden in developing countries. The aim of this study was to determine the feasibility of mobile health (mHealth) phone technology to improve management and follow-up of clients with cervical cancer precursor lesions.

**Methods:**

A sequential mixed methods design was employed for this study. Quantitative data was collected using a cross-sectional survey of 364 women eligible for a Pap smear at public sector health services in Cape Town, South Africa. Information was collected on socio-demographic status; cell phone ownership and patterns of use; knowledge of cervical cancer prevention; and interest in Pap smear results and appointment reminders via SMS-text messages. Descriptive statistics, crude bivariate comparisons and logistic regression models were employed to analyze survey results. Qualitative data was collected through 10 in-depth interviews with primary health care providers and managers involved in cervical cancer screening. Four focus group discussions with 27 women attending a tertiary level colposcopy clinic were also conducted. Themes related to loss of mobile phones, privacy and confidentiality, interest in receiving SMS-text messages, text language and clinic-based management of a SMS system are discussed. Thematic analyses of qualitative data complemented quantitative findings.

**Results:**

Phone ownership amongst surveyed women was 98% with phones mostly used for calls and short message service (SMS) functions. Over half (58%) of women reported loss/theft of mobile phones. Overall, there was interest in SMS interventions for receiving Pap smear results and appointment reminders. Reasons for interest, articulated by both providers and clients, included convenience, cost and time-saving benefits and benefits of not taking time off work. However, concerns were expressed around confidentiality of SMS messages, loss/theft of mobile phones, receiving negative results via SMS and accessibility/clarity of language used to convey messages. Responsibility for the management of a clinic-based SMS system was also raised.

**Conclusions:**

Results indicated interest and potential for mHealth interventions in improving follow-up and management of clients with abnormal Pap smears. Health system and privacy issues will need to be addressed for mHealth to achieve this potential. Next steps include piloting of specific SMS messages to test feasibility and acceptability in this setting.

## Background

Cervical cancer, the fourth commonest cause of cancer deaths in women worldwide, remains a global public health challenge [1]. In South Africa (SA), it is the second most common cancer and the leading cause of cancer mortality in women [2, 3]. Moreover, South Africans continue to face one of the worst human immunodeficiency virus (HIV) epidemics in the world with approximately one fifth of South African woman between 15 and 49 years currently living with the disease [4]. These women are at increased risk for human papillomavirus (HPV) infection, the underlying cause of cervical cancer; cervical precancerous lesions and; cervical cancer [5, 6]. Without adequate screening and treatment of cervical precancerous lesions, the benefits of an expanded antiretroviral program in this context will likely by offset by an increased burden of cervical cancer.

A Papanicolaou (Pap) smear has long been regarded as an effective cytology-based screening tool to detect precancerous cervical abnormalities [7]. Further investigation of these abnormalities typically includes assessment with a magnifying instrument (a colposcope) that enables good visualization of the cervix. When clients with abnormal Pap smears receive the necessary screening, investigation and treatment, cervical cancer can be prevented. In developed regions, organized cervical screening programs supported by well-functioning recall systems have resulted in reduced cervical cancer incidence and mortality [7, 8]. However, in developing countries, cervical cancer remains a persistent concern due to ineffective or absent screening programs, lack of awareness, health system challenges and resource constraints [7, 9–11].

In SA, women over 30 years of age attending public health facilities are eligible for three free Pap smears in their lifetime [12]. Women who are HIV positive are eligible for a Pap smear at diagnosis and every 3 years if the screening test is negative. Women with abnormal Pap smear results are referred for a colposcopy assessment. Pap smear screening services are available at most primary healthcare facilities, while colposcopies are typically conducted at tertiary or secondary level hospitals. Typically, clients are informed of their colposcopy appointments via postal recall system sent directly from the laboratory or through their primary health care clinic of origin.

A key challenge to successful implementation of cervical screening programs in SA has been the loss to follow-up of women with abnormal Pap smears at colposcopy appointments. A study on the challenges faced by these women across three provinces in SA indicated that, although appointments were made for all women with high-grade squamous intraepithelial lesions, only 50% of the women attended [13]. Poor attendance occurred despite improvements in interfacility linkages and established paper-based feedback systems.

Various strategies and interventions in several countries have been shown to improve colposcopy attendance and screening program effectiveness, including the use of reminder telephone calls and provision of travel incentives [14]. In SA, repeated home visits by community health workers have also shown some efficacy [15]. However, these interventions are costly, time-consuming and place additional burdens on clinic staff. More recently, investigators have raised the potential of mobile phone-based interventions, including the use of short message service (SMS), as a cost-effective alternative to delivering Pap smear results, improving waiting times and providing information [16].

Mobile phones are one of the fastest growing technologies globally with over five billion unique mobile subscriptions to date [17]. In SA, mobile phones have reportedly penetrated 98% of households, reaching more people than public water and sanitation services [18]. Its near ubiquitous use makes it a compelling tool for improving health service delivery and bridging systematic gaps in access to care [19–21]. Mobile health (mHealth), a broad term used to describe the use of mobile communication technologies for the delivery and support of health care, is gaining increasing attention [22, 23]. Several studies have described the feasibility and benefits of mHealth technology in SA to conduct research, health promotion, adherence support and management of community-based social and health services [24–30]. The use of mobile phones, specifically SMS text messaging, is also becoming common for preventative health care [31]. However, there remains a shortage of evidence to support its use in cancer prevention, especially in developing contexts [32]. Against a backdrop of growing access to, and increasing enthusiasm for, the use of mHealth, this study explored the feasibility of using mobile phones to improve the management and follow-up of clients with cervical cancer precursor lesions in Cape Town, SA.

## Methods

A sequential mixed methods design was employed for this study. Quantitative data was collected first using a cross-sectional survey of women eligible for a Pap smear at public sector health services in Cape Town. Qualitative data complemented the interpretation of quantitative data and was collected through in-depth interviews (IDIs) with primary health care providers and managers and focus group discussions (FGDs) with first time colposcopy clinic attendees.

### Quantitative data

The cross-sectional survey was conducted between February and June 2014 among women eligible for a pap smear. The study sample was stratified by age. Women between 18 and 30 years (*n* = 187) were recruited from 2 HIV care clinics and women ≥30 years (*n* = 177) recruited from 2 primary health care clinics. Surveys were administered verbally by a trained researcher in the home language of participants (English, Afrikaans or isiXhosa). Information was collected on socio-demographic status; cell phone ownership and patterns of use; knowledge of cervical cancer prevention; and interest in Pap smear results and appointment reminders via SMS-text messages using a structured questionnaire. Data was analyzed using STATA. Descriptive statistics were used to characterize the variables and crude bivariate comparisons used to identify associations between patterns of cell phone use, socio-demographics and other variables. Bivariate comparisons were made using parametric tests for categorical data (either chi-squared or Fisher exact where *n* < 5 in a particular cell) or non-parametric tests such as the Kruskal-Wallis test for data that were not normally distributed. Logistic regression models were employed to determine factors associated with interest in receiving Pap smear results and interest in receiving appointment reminders via SMS. Variables entered into the models included those with *p* <  0.05 on bivariate analysis and those of a priori interest. Where multicollinearity was present, we included variables in the model that minimized the negative log likelihood. HIV status and age showed collinearity as anticipated given our recruitment process. We excluded age, but included HIV status in the final model. The model for interest in receiving Pap smear results via SMS included education level (less than matric vs. matric or higher), reported HIV status, likelihood of SMS being seen by others and past Pap smear. For interest in receiving appointment reminders via SMS, the model included education level (less than matric vs. matric or higher), reported HIV status, likelihood of SMS being seen by others, past Pap smear type and whether the participant spoke Afrikaans.

### Qualitative data

IDIs (*n* = 10) were conducted with primary health care providers and managers between June and August 2014. A researcher trained in qualitative interview techniques used a semi-structured interview guide to elicit views on the use and feasibility of mobile technology to improve management of the cervical cancer screening program. Additionally, four focus group discussions (FGDs) were conducted with first-time colposcopy clinic attendees (18 years and older) at a tertiary hospital in Cape Town in November 2014. Discussions lasted between 90 and 120 min and were conducted predominantly in English, with Afrikaans and isiXhosa translation provided where necessary. The following issues were explored by a trained interviewer: challenges to colposcopy clinic attendance; potential use of mobile technology to improve adherence; and content of SMS-text messages. The interviewer kept detailed reflective notes during the IDIs and FGDs, which were used in interpreting the results. Both IDI and FG discussions were digitally-recorded, translated (where necessary) and transcribed. Transcripts were reviewed by JM and MM. Data were entered into QSR NVivo 10 (a qualitative computer software package). A thematic analysis was performed with initial coding categories for analyzing data drawn from the interview guides. MM coded the data and JM reviewed the coded data. Text segments were coded and categorized into basic themes.

Written informed consent was obtained from all participants. Ethical approval to conduct the study was obtained from the Human Research Ethics Committee, University of Cape Town.

## Results

The following section presents the profile characteristics of each sample group followed by a narrative discussion of integrated quantitative and qualitative findings.

### Participant profiles

#### Cross-sectional survey among women eligible for a pap smear

A total of 412 women were approached to participate in the survey. One declined and 40 did not meet the age eligibility criterion. Of the remaining 371 participants, 364 (98%) owned a mobile phone. Results presented are based on the 364 participants (187 <  30 years and 177 ≥ 30 years) who reported current mobile phone ownership. The main home language spoken was isiXhosa, however the majority of participants (87%) also spoke English (Table 1). Most participants had a secondary or higher level of education. Among women older than 30 years, 97% (171) were aware of their HIV status. Of these 97% were prepared to disclose their status to the interviewer and 55% were HIV positive.

**Table 1:** Profile of participants who currently owned a mobile phone

Characteristic	< 30 years (*n* = 187)	≥ 30 years (*n* = 177)	Total (*N* = 364)	*P*-value^a^
Median age in years (IQR)	25 (23–28)	37 (32–42	29 (25–36)	
Main home language				
Afrikaans	13 (7%)	21 (11.9%)	34 (9.3%)	0.272
English	1 (0.5%)	3 (1.7%)	4 (1.1%)	
isiXhosa	165 (88.2%)	145 (81.9%)	310 (85.2%)	
Other	8 (4.3%)	8 (4.5%)	16 (4.4%)	
Languages spoken (after main home language)				
Afrikaans	29 (15.5%)	45 (25.4%)	74 (20.3%)	0.019
English	158 (84.5%)	132 (74.6%)	315 (86.5%)	0.012
isiXhosa	174 (93.1%)	150 (84.8%)	324 (89.0%)	0.011
Other	25 (13.4%)	27 (15.3%)	52 (14.3%)	0.607
Highest level of education				0.007^b^
Primary or less	16 (8.6%)	35 (19.8%)	51 (14.0%)	
Secondary	167 (89.3%)	137 (77.4%)	304 (83.5%)	
Post-secondary	4 (2.1%)	5 (2.8%)	9 (2.5%)	
Formal housing	83 (44.4%)	81 (45.8%)	164 (45.1%)	0.792
Median number of people living in home (IQR)	4 (3–6)	4 (3–5)	4 (3–6)	0.029^c^
Employed	52 (27.8%)	81 (45.8%)	133 (36.5%)	< 0.001

(*IQR* Interquartile range)

^a^P-value for Chi-squared test unless otherwise stated

^b^Fishers exact test

^c^Kruskal-Wallis test

#### In-depth interviews with health care providers

Interviews were conducted until data saturation was reached. In total we interviewed three primary health care clinic managers and seven nurse practitioners involved in administering Pap smears. The majority of providers were female (*n* = 8) and six were younger than 40 years. On average, providers were involved in the Pap smear screening program for 3 years (range of 1–10 years).

#### Focus group discussions with colposcopy clients

Twenty-seven first time colposcopy clients participated in four focus group discussions (6 or 7 participants per group). The median age of participants was 34 years (IQR 27–40 years). Eighteen out of 27 (67%) had a secondary school or higher level of education and 15/27 (56%) were unemployed. Twenty-four participants (89%) owned a mobile phone.

During the qualitative IDIs and FGDs themes related to mobile phones loss, privacy and confidentiality, interest in receiving SMS-text messages, text language and clinic-based management of a SMS system emerged and are presented in this paper. Themes relating to women’s experiences with cervical cancer screening also emerged during the FGDs and have been discussed elsewhere [9].

### Mobile phone ownership and patterns of phone use among women eligible for a pap smear

Information on ownership and patterns of use were obtained as part of the cross-sectional survey and are presented in Table 2. Younger women were significantly more likely to own a smart or feature phone compared to older women (68% vs. 55%, *p* = 0.001). The majority of participants (97.9%) owned their current subscriber identity module (SIM) card, with 16% owning two or more SIM cards. Overall use of mobile phones to make or receive an SMS was high. Younger respondents were significantly more likely to make use of the SMS function, use WhatsApp (a cross-platform mobile messaging application that uses the internet for sending text messages, images and videos) and access the internet, compared to older respondents.

**Table 2:** Mobile phone ownership and patterns of use

Characteristic	< age 30 (*n* = 187)	≥ age 30 (*n* = 177)	Total sample (*N* = 364)	*P*-value^a^
Mobile phone type				
Basic	60 (32.1%)	68 (38.4%)	128 (35.2%)	0.001
Smart/Feature	127 (67.9%)	98 (55.4%)	225 (61.81%)	
Unknown	0 (0%)	11 (6.21%)	11 (3.0%)	
Cash purchase of phone (as opposed to contract)	177 (94.7%)	169 (95.5%)	346 (95.1%)	0.716
Median time owned mobile phone (IQR)	12 months (5–24)	8 months (6–36)	12 months (5–30)	< 0.001
Ever lost or had mobile phone stolen	115 (61.5%)	95 (53.7%)	210 (57.7%)	0.031
Someone else has access to phone	99 (52.9%)	84 (47.5%)	183 (50.3%)	0.296
Owns SIM card	183 (97.9%)	174 (98.3%)	357 (98.1%)	0.758
Ever uses someone else’s SIM card	12 (6.4%)	8 (4.5%)	20 (5.5%)	0.427
Ways phone is used				
Receive SMS	187 (100%)	170 (96.1%)	357 (98.1%)	0.006^b^
Send SMS	186 (99.5%)	165 (93.2%)	351 (96.4%)	0.001^b^
Make calls	187 (100%)	177 (100%)	364 (100%)	–
Receive calls	187 (100%)	175 (98.9%)	362 (99.5%)	0.236^b^
Send money	13 (7%)	14 (7.9%)	27 (7.4%)	0.727
Receive money	15 (8%)	16 (9%)	31 (8.5%)	0.728
WhatsApp	103 (55.1%)	67 (37.9%)	170 (46.7%)	0.001
Mxit	35 (18.7%)	21 (11.9%)	56 (15.4%)	0.070
Browse internet	84 (44.9%)	51 (28.8%)	135 (37.1%)	0.001
Send/receive emails	36 (19.3%)	21 (11.9%)	57 (15.7%)	0.053

^a^P-value for Chi-squared test unless otherwise stated

^b^Fishers exact test

### Theft or loss of mobile phones

Although most health care providers were in favour of using an SMS to convey Pap smear results and for colposcopy appointment reminders, many were concerned that the SMS might not reach the intended client because of potential loss or theft of mobile phones.

*“There could be challenges … you just send the SMS, so the results might end up landing with the wrong person and maybe the phone got lost or stolen.” (*Health manager, > 60 years old)

The perceived high rate of mobile phone loss or theft was confirmed in the cross-sectional survey. Overall 58% of survey respondents had ever experienced mobile phone loss or theft (Table 2), with 28% of all respondent having experienced mobile phone loss or theft in the past year. During the FGDs with colposcopy clients some suggested that to overcome problems of theft or loss, the SMS should also be sent to a person that the client trusts (e.g. close family or friend) and this person could then forward the SMS to the intended recipient.

*“Yes, the SMS is okay - however later your phone is lost or stolen. I think it would be better if they can also take contact details of a family member you trust so that they can also send the SMS to him/her. In that way that person will bring the notification to your attention.”* (FGD3)

### Confidentiality

Some health care providers were concerned that communicating via SMS could breach confidentiality because of possible sharing of mobile phones in the communities they served.

*“Many of our patients are not working and cannot afford a cell phone… Now, because not everybody owns a cell phone, they share it, sometime leave with a neighbor to charge the battery and the fear is confidentiality as other people might see the SMS.”* (Clinical nurse practitioner, 40–50 years old)

Half of the cross-sectional survey respondents and the majority of the FGD participants (85%) indicated that someone else had access to their phone, usually a family member. Some FGD participants raised concerns about an SMS from the clinic being seen by someone else.

*“I don’t like messages sent to my phone, sometimes you leave your phone on a charger and a message comes in, someone picks it up and reads without your permission.”* (FGD4)

### Receiving pap smear results via SMS

Most (72%) survey respondents were interested in receiving Pap smear results by SMS. Reasons for interest included convenience, cost and time saving and the benefit of not needing to take time off work. These views were echoed by colposcopy clients.

*“Getting a SMS is very good because now you know for sure that your Pap smear results are available, instead of being given a return date only to find out when you get there your results have not yet been received. Remember, we are working and we take time off work to come to the clinic.”* (FGD2)

*“Sometimes SMS is better than calls because sometimes when a hospital calls you whilst you are busy and can’t pick up the phone and they will not call you again to give you the results.”* (FGD4)

Health care providers also suggested that the SMS system would be more convenient for women and an efficient use of resources.

*“I think that for me it could be a very good thing* [using SMS] *that is also convenient for the patient because nowadays patients are becoming well informed health-wise because they themselves will come and ask you, maybe they did a Pap smear last week, saying “sister are my Pap smear results not yet available”. They are also concerned about their health status which is a beautiful thing.”* (Clinical nurse practitioner, 40–50 years old)

*“I would recommend or support that the SMS system … I mean if we can manage time well and also optimal utilization of resources, the SMS will instantly reach the patient and she will receive the results.”* (Health manager, 50–60 years old)

Results of the logistic regression analysis on factors associated with interest in receiving Pap smear results via SMS are summarized in Table 3. Women with higher education level and a previous Pap smear were significantly more interested in receiving results via an SMS (adjusted odds ratio (aOR) 2.15 (aOR) and 1.78 (aOR), respectively). Respondents were significantly less likely to want to receive Pap smear results via SMS if someone else had access to their mobile phone (aOR 0.31, 95% CI 0.18–0.51). Although HIV positive women were less interested in receiving Pap smear results via an SMS compared to HIV negative women, this difference was not statistically significant.

**Table 3:** Factors associated with interest in receiving Pap smear results via SMS

Variable	Adjusted OR (95% CI)^a^	*P*-value
Uses text	4.34 (0.54–34.94)	0.168
Completed high school	2.15 (1.16–3.98)	0.015
Previous pap smear	1.78 (1.04–3.07)	0.037
Phone not private	0.31 (0.18–0.51)	< 0.001
HIV positive	0.65 (0.34–1.25)	0.196

^a^Adjusted by all the variables reported in the table

Most FGD participants mentioned it would be acceptable to convey normal results via SMS, but not abnormal results. In the event of abnormal results, participants generally felt the message should simply read: “please come to the clinic.”

*“If the results are okay they can let me know that I don’t need to worry my results are normal however if there is something wrong I would prefer that they do not put it in the SMS rather send SMS asking me to come to the clinic so that they can explain everything to me.”* (FGD2)

Many health care providers were also concerned about clients being given abnormal results outside the clinic environment, preferring face-to-face discussions.

*“So when I give the results via SMS there is no one to explain to them what the results mean”* (Professional nurse, < 30 years old)

*“For some, if you say maybe there is a lesion, immediately they think of cancer and get so worked up but if you interpret and tell the patient what is actually happening to relieve that anxiety you are bound to get a positive result and they will likely honor the appointment scheduled for them…Now I’m not sure if you can put all that in an SMS to alleviate the patient from any anxiety she might have.”* (Clinical nurse practitioner, 40 – 50 years old)

### Receiving appointment reminders via SMS

The majority of survey respondents (77%) were interested in receiving clinic or hospital appointment reminders. However, HIV positive women were 0.3 times less interested in receiving appointment reminders via SMS compared to HIV negative women (aOR 0.33, 95%, CI 0.15–0.75), whilst women who spoke Afrikaans were significantly more interested in receiving appointment reminders (aOR 3.01, 95% CI 1.23–7.37) (Table 4).

**Table 4:** Factors associated with receiving appointment reminders via SMS

Variable	Adjusted OR (95% CI)^a^	*P*-value
Uses text	14.19 (1.72–117.13)	0.014
HIV positive	0.33 (0.15–0.75)	0.008
Afrikaans speaking	3.01 (1.23–7.37)	0.016
Phone not private	0.83 (0.49–1.41)	0.495
Complete high school	1.26 (0.70–2.27)	0.435
Previous Pap smear	0.80 (0.44–1.45)	0.455

^a^Adjusted by all variables reported in the table

FG participants reported waiting for up to 7 months for a colposcopy appointment. The majority indicated interest in receiving appointment reminders, with most preferring to receive two reminders, one soon after the colposcopy appointment is made and a second between 1 to 7 days before the appointment date.

Health care providers were in favour of sending clients appointment reminders via SMS for similar reasons articulated by FGD respondents.

*“Usually the appointment is in four weeks, so nearer to the time you can send an appointment reminder. Others do forget genuinely, you see them when they come to the clinic* [primary health care clinic] *whereas they were supposed to attend the colposcopy clinic. I really think it would work to the patient’s advantage.”* (Clinical nurse practitioner, 40 – 50 years old)

### Language

Some cross-sectional survey respondents did not want to receive Pap smear results via SMS because of potential difficulty in understanding the message. Both FG discussants and health care providers emphasized the importance of using simple written language and highlighted potential difficulties with use of medical terminology.

*“They can say anything as long as they use simple easy to follow language.”* (FGD3)

*“I prefer it to be in English because there are some words that cannot be communicated in isiXhosa. Sometimes it becomes difficult to read medical terms on isiXhosa.”* (FGD3)

*“...in a language that would be understood by the client.”* (Clinic manager, 30 – 40 years old)

### Management of a clinic-based SMS system

All health care providers were familiar with using mobile phones and some had used their personal phones to contact clients via text.

*“We* [practitioners] *do not have access to call cell phones* [from the clinic landline] *… I call them using my* [personal] *cell phone and sometimes send a message* [SMS]...” (Professional nurse, 30–40 years old)

However, healthcare providers had no experience of a clinic-based SMS system. Some were unaware that SMS messages could be computer generated and suggested that clinics should have dedicated mobile phones for SMS communication with clients.

In discussion on how a clinic-based SMS system could work, health care providers recommended the following: confirming phone numbers at each clinic visit, clients be given an option of whether they wanted to receive SMS notification of results, and that clients are informed as to when they can expect the SMS notification. Some felt that only clients with abnormal results be sent an SMS, and by implication clients that did not receive an SMS would interpret this as having a normal Pap smear result. Others were concerned about non-receipt of the SMS and preferred that clients be asked to confirm receipt. Some suggested that clients be instructed that if an SMS is not received within a defined period, then they should return to the clinic.

*“The nurse will also confirm the phone numbers…of the patient and ask the patient if it would be okay to phone them. So, they have got an arrangement with the clients, that says so if you do not get an SMS from me you must know your result is okay but if you do receive an SMS from me then you must know that you must come to the clinic for your results.”* (Operational manager, > 60 years old)

There were mixed views on who should be responsible for managing a clinic-based SMS system. Some felt that the health care provider performing the Pap smear should be responsible for sending the SMS as a relationship had already been established between the provider and client and that administrative staff would not understand the urgency of contacting clients and ensuring that clients responded.

*“The cervical smear portfolio holder* [should be responsible for sending the SMS]*, because she is the one in contact with the clients because she is the one performing, taking smears from the patients and teaching and educating them about the smears. So that relationship needs to continue and the patient has already established a relationship with that person*.” (Clinic manager, 30–40 years)

Others felt that it would be an additional burden on the health care providers’ already heavy workload, and that the task should be the responsibility of the administrative staff. A few suggested it should be the responsibility of the health educators.

## Discussion

The use of mhealth in the management of communicable and non-communicable diseases is increasing [32–38] with some evidence that SMS interventions could improve treatment adherence, increase appointment attendance and support behavior change [39–45]. However, limited research is available on the feasibility of mhealth in the control and management of cervical cancer in low- and middle-income countries (LMICs) [32]. Consequently, there is an important need for formative research to guide the development and implementation of mhealth interventions in these settings.

This study showed that the vast majority of women eligible for a Pap smear at public sector primary health care clinics in SA owned a mobile phone. Additionally, women were familiar with sending and receiving SMS text messages, making mobile phone technology a potential mechanism to improve follow-up and management in a cervical cancer screening program. Both women and health care providers were interested in using mobile phones to convey Pap smear results, with most preferring the SMS text to indicate availability rather than the actual result, particularly for abnormal smears.

In countries where access to a fixed-line telephone is limited, the mobile phone is rapidly becoming an important means of sending appointment reminders [46]. Missed colposcopy appointments are a major barrier to cervical cancer prevention and treatment in SA. In our study there was considerable support for an SMS colposcopy clinic appointment reminder system. Given the long waiting time for a colposcopy appointment in the public-sector health system, it is not surprising that participants suggested a minimum of 2 reminders, with the 2nd being closer to the appointment date.

Our research also highlighted several issues that need to be addressed in designing an mhealth intervention to improve Pap smear management. Similar to Crankshaw et al. [36] participants in our study reported a high loss of mobile phones (58% had ever lost a mobile phone, 28% in the preceding year). This has implications for the success and sustainability of any mhealth intervention. Although participants suggested regular updating of mobile phone numbers at clinic visits as a possible solution to the high mobile phone turnover, this has limited value for cervical cancer screening clients who typically are asymptomatic and therefore unlikely to visit the clinic regularly. Another solution is for mHealth interventions to make provision for clients to send a free confirmation of receipt of an SMS. This will assist clinic managers in identifying those that did not receive the text message and therefore require follow-up via other clinic client-tracking systems such as via community health workers.

Previous studies have highlighted the importance of privacy with SMS based interventions [36, 46, 47]. In our study many clients reported that others had access to their mobile phones, with concerns about confidentiality raised by both providers and clients. Clients who reported that others were likely to have access to their messages were significantly less interested in receiving Pap smear results via SMS. HIV positive clients were also significantly less interested in receiving appointment reminders, possible due to privacy concerns. In designing an intervention, providers would need to discuss the possibility of breaks in confidentiality and confirm clients’ interest in receiving text messages before enrollment. Interventions that require a password to access messages could provide privacy, however client trust in such interventions will need to be explored.

Difficulty in understanding text messages could be a potential barrier to uptake of an SMS intervention [34]. Our results concur with others on the importance of careful development of text messages [48, 49], with attention to use of simple, clear language and accurate translation into local languages. Careful piloting of specific mobile text messages with assessment of understanding and acceptance of messages will also be required.

Any mhealth intervention will need to carefully consider integration into existing and developing public sector health information and management systems [20, 34, 50, 51]. Mhealth interventions will be greatly facilitated by electronic patient clinic records and electronic appointment booking systems [50]. Currently this is largely unavailable in the SA public health system. Health care providers have limited experience of a clinic-based SMS system and will require information and training. Issues related to roles and responsibilities in terms of management of a SMS intervention were raised and coherent policy and guidelines will need to be developed. In addition, development of relevant data security policy that protects client information during message transmission and data storage will be also critical as the mHealth field of develops. The current lack of supporting policy has been identified as an important barrier to implementation of mhealth interventions worldwide [46, 50].

As our study was conducted in an urban setting where mobile phone ownership is higher compared to rural areas (85% vs. 68%), findings are unlikely to be generalizable to rural areas [52]. A limitation of our study was that all client interviews were conducted at health facilities. Although clients were informed that their participation and responses would not affect the care received, it is possible that some degree of courtesy bias could have resulted in an overestimate of client willingness to receive Pap smear results and appointment reminders via SMS. Detailed reflective notes were kept during focus group discussions and contributed to interpretation of findings, however interactions between participants were not analyzed.

## Conclusion

Results from this study demonstrate that mHealth interventions, specifically the use of SMS text-based messaging, are perceived positively by the majority of the women interviewed. Such interventions may tackle health system inefficiencies in the communication of test results and appointment attendance. However, important issues, including confidentiality and privacy concerns as well as the integration and management of mHealth interventions into the broader health information system, will need to be addressed for this potential to be realized.

## Abbreviations

aOR: Adjusted odds ratio
FGD: Focus group discussion
HPV: Human papillomavirus
IDI: In-depth interview
IQR: Interquartile range
mHealth: Mobile health
Pap smear: Papanicolaou smear
SA: South Africa
SMS: Short message service
